# Development of immortalized mouse aortic endothelial cell lines

**DOI:** 10.1186/2045-824X-6-7

**Published:** 2014-04-01

**Authors:** Chih-Wen Ni, Sandeep Kumar, Casey J Ankeny, Hanjoong Jo

**Affiliations:** 1 Wallace H. Coulter Department of Biomedical Engineering Georgia Institute of Technology and Emory University, 1760 Haygood Drive, Health Science Research Building, E-170, Atlanta, GA 30322, USA; 2 Division of Cardiology, Emory University, 1760 Haygood Drive, Health Science Research Building, E-170, Atlanta, GA 30322, USA; 3 Department of Biomedical Engineering, Khalifa University of Science, Technology and Research (KUSTAR), PO BOX 127788, Abu Dhabi, UAE

**Keywords:** MAEC, Endothelial cells, Shear stress, p47phox, eNOS, cav1

## Abstract

**Background:**

The understanding of endothelial cell biology has been facilitated by the availability of primary endothelial cell cultures from a variety of sites and species; however, the isolation and maintenance of primary mouse aortic endothelial cells (MAECs) remain a formidable challenge. Culturing MAECs is difficult as they are prone to phenotypic drift during culture. Therefore, there is a need to have a dependable *in vitro* culture system, wherein the primary endothelial cells retain their properties and phenotypes.

**Methods:**

Here, we developed an effective method to prepare immortalized MAEC (iMAEC) lines. Primary MAECs, initially isolated from aortic explants, were immortalized using a retrovirus expressing polyoma middle T-antigen. Immortalized cells were then incubated with DiI-acetylated-low density lipoprotein and sorted via flow cytometry to isolate iMAECs.

**Results:**

iMAECs expressed common markers of endothelial cells, including PECAM1, eNOS, VE-cadherin, and von Willebrand Factor. iMAECs aligned in the direction of imposed laminar shear and retained the ability to form tubes. Using this method, we have generated iMAEC lines from wild-type and various genetically modified mice such as p47^phox-/-^, eNOS^-/-^, and caveolin-1^-/-^.

**Conclusion:**

In summary, generation of iMAEC lines from various genetically modified mouse lines provides an invaluable tool to study vascular biology and pathophysiology.

## Introduction

Extensive research supports the notion that several common vascular diseases are, in part, a consequence of endothelial responses to shear stress from blood flow; i.e., that prolonged endothelial activation leads to dysfunction, which is an early, preclinical component of vascular disease [1,2]. Unfortunately, it is difficult to access vascular tissue directly *in vivo* and sequentially during these preclinical stages of disease development; without such tissue, the endothelial cell’s contribution to disease development can only be deduced. As a consequence, most research in vascular biology continues to (1) focus on the footprints of disease by analyzing damaged endothelium; (2) link putative circulatory factors to disorders through their effect on cultured ECs, often derived from unaffected tissue; and (3) develop animal models that may simulate human diseases. Moreover, endothelial dysfunction is thought to be one of the earliest stages in the onset of atherosclerosis [3]. This dysfunction is characterized by gene dysregulation and inflammatory responses [3,4]. Therefore, *in vitro* EC cultures are important tools for studying vascular physiology and disease pathology.

EC from different origins and species have been successfully cultured for several decades [5,6]. The most common human primary ECs used in culture are human umbilical cord vein endothelial cells (HUVEC) [7], human aortic endothelial cells (HAEC) [8], human coronary artery endothelial cells (HCAEC) [9], and microvascular ECs [10,11]. In addition, ECs have been isolated from various species, such as bovine aortic endothelial cells (BAEC) [12], pig aortic endothelial cells (PAEC) [13] and mouse EC [3,14-27]. Due to numerous transgenic mouse lines, the isolation and culture of mouse ECs is of particular interest. Several studies have developed methods for isolation of primary mouse aortic endothelial cells (MAEC) for *in vitro* study [18,19,22-27]; however, the isolation and maintenance of primary MAEC continues to be challenging and time-consuming, cost-consuming, and labor-intensive. The main obstacles in primary MAEC isolation include low cell numbers from individual mice, limited proliferative potential of the cells, and contamination with other cell types. Moreover, studies have shown MAEC have a great propensity to transdifferentiate to mesenchymal cells during culture [28]. Therefore, development of stable, immortalized MAEC lines that retain the characteristics of endothelial cells would greatly facilitate endothelial biology and pathology research.

In this study, we have developed an effective method that enabled us to generate several iMAEC lines, including iMAEC from wild-type mice (iMAEC-WT), eNOS knockout mice (iMAEC-eNOS), caveolin-1 knockout mice (iMAEC-cav), and p47^phox^ knockout mice (iMAEC-p47). We carried out extensive characterization to confirm that these iMAEC lines maintain endothelial phenotype and functional characteristics during culture.

## Methods

### Mice

Mouse aortic endothelial cells (MAEC) were isolated from the thoracic and abdominal aortas of various control and knockout mouse lines. Wild-type C57Bl/6 and p47^phox^ knockout mice were purchased from Jackson Laboratories (Bar Harbor, Maine). Caveolin-1 (cav1) knockout mice were kindly provided by Dr. Marek Drab (Max Planck Institute for Molecular Cell Biology and Genetics, Dresden, Germany). eNOS knockout mice were kindly provided by Dr. Mark C. Fishman and Dr. Paul Huang (Cardiovascular Research Center, Harvard Medical School, Charlestown, MA). All animals were maintained according to the approved Institutional Animal Care and Use Committee protocol by Emory University.

### Primary MAEC isolation

**Figure 1:**
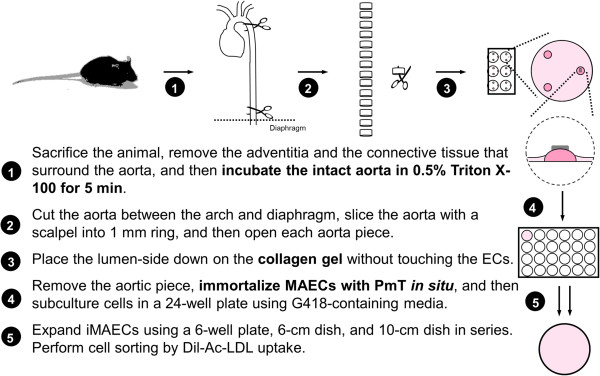
Scheme of mouse aortic endothelial cell isolation and immortalization.

Initially, 4-week-old male mice were used for MAEC isolation. Each mouse was sacrificed by CO_2_ asphyxiation and cleaned using 70% ethanol. The abdominal and thoracic cavities were opened and the mouse was perfused, via the left ventricle, with 3-4 mL of sterile heparinized (10 U/mL) 1X Hank's buffered salt solution (HBSS, Cellgro). Most organs were removed except for the thoracic/abdominal aorta, which was left intact. Perivascular fat and adventitia were removed from the ventral side of the aorta. To decrease contamination of medial smooth muscle cells, the cleaned aortas were perfused with HBSS containing 0.5% Triton X-100 for 5 minutes. The aorta was then dissected out, rinsed 5 times with fresh HBSS, and placed in a sterile dish of cold HBSS. The aorta was then cut into small rings (~1 mm length) using a sterile scalpel. Then, each aortic ring was opened and was carefully laid on a collagen gel bead with the endothelial cells directly facing the gel. The collagen gel consisted of type I collagen (Bio-Rad) diluted with EGM2-MV (Lonza) to a final concentration of 1.75 mg/mL. Each collagen gel bead was prepared using 20 µl collagen and was allowed to solidify at 37°C for at least 30 min before use. Note that each aortic piece were positioned flat on the surface of the collagen gel. After tissue placement, the gel bead and aortic piece were kept hydrated with EGM2-MV and care was taken to avoid completely submerging and dislodging the aorta piece from the bead. The explants were cultured at 37°C and 5% CO_2_ in an incubator and monitored daily. The key steps of primary MAEC isolation are summarized in Figure 1.

### Immortalization

Since polyoma middle-sized T-antigen (PmT) is known to specifically immortalize endothelial cells [29,30], we used the same method to immortalize MAEC as previously described by Balconi et al. to generate ECs from embryonic stem cells [31]. A PmT-producing packaging cell line was kindly provided by Dr. Elisabetta Dejana (Institute of Pharmacological Research, Milan, Italy). Briefly, PmT-conditioned medium was collected, filter sterilized using a 0.22 μm filter, and stored at -80°C until use. Once aortic cells began to grow out of the explants on the collagen gel, the aorta piece was removed within the next 3 to 4 days and complete growth medium (DMEM with 10% FBS, 1% endothelial cell growth supplement (ECGS) crude extract, 1% penicillin and streptomycin) was added. After one day of culture, MAEC were treated with the preserved PmT-conditioned medium, along with 8 μg/mL polybrene to increase infection efficiency (Sigma) for 4 hours at 37°C. After PmT incubation, the PmT-conditioned medium was removed and replaced with complete growth medium. 48 hours later, cells were passaged into a 24-well plate and grown in growth medium containing G418 (800 μg/mL) to select for immortalized cells containing the neomycin resistance gene. Cells were observed and passaged for several weeks (4 to 8 weeks) before complete cell selection was observed.

### FACS cell sorting

Cells were stained with acetylated low density lipoprotein (Ac-LDL), labeled with 1,1′-dioctadecyl– 3,3,3′,3′-tetramethyl-indocarbocyanine perchlorate (DiI-Ac-LDL, Biomedical Technologies) and then sorted by fluorescence-activated cell sorting. Briefly, cells were incubated with 10 μg/mL DiI-Ac-LDL for 4 hours at 37°C. Cells were then washed three times with fresh growth medium, trypsinized with 0.05% trypsin-EDTA, pelleted for 3 minutes at 2,300 RPM, and resuspended in 0.5-1.0 mL of sterile sorting buffer (1% FBS in 1X calcium- and magnesium-free HBSS). iMAEC were then sorted using a FACS Vantage SE (Becton Dickinson, San Jose, CA) using common gates for morphology (FSC-H vs SSC-H), singlets (FSC-W vs SSC-H), and separation gates for DiI staining. Since our goal was to collect pure iMAECs without any other contaminating cell types, we applied a highly stringent positive gating strategy by collecting only those cells that exhibited high DiI-LDL fluorescence intensity, which resulted in >95% DiI-positive cells post sorting. To effectively establish a stringent gating strategy, HUVECs and rat aortic smooth muscle cells (kindly provided by Dr. Kathy Griendling) were used as positive and negative controls, respectively. DiI-positive cells were collected in complete growth media and seeded on to a gelatin-coated (0.1%) culture dish. iMAEC were then observed and passaged at a ratio of 1 to 2 until characterization experiments.

### Immunocytochemistry

Primary antibodies against PECAM-1 (Santa Cruz), VE-Cadherin (Cayman Chemical), von Willebrand Factor (WF) (Dako), and smooth muscle alpha actin (α-SMA, Sigma) were used for immunocytochemical staining of iMAEC and control cells, namely HUVEC as a positive control and 3T3 fibroblast and rat aortic smooth muscle cells (RASMC) as negative controls. Cells were fixed with 4% paraformaldehyde and permeabilized in 0.2% Triton X-100. Primary antibody diluted in 3% bovine serum albumin was applied overnight at 4°C, followed by incubation with a secondary antibody conjugated rhodamine red-X (Molecular Probes) for 1 hour at room temperature. Nuclei were labeled with Hoechst #33258 diluted in 3% bovine serum albumin for 15 min at room temperature. All cells were mounted using Dako mounting media (Dako), and fluorescence images were captured using fluorescence microscopy (Zeiss epi-fluorescent microscope).

### Shear stress studies

iMAEC were grown to confluency in 100-mm tissue culture dishes (Falcon) and were subsequently exposed to laminar shear (LS, 15 dynes/cm^2^) or oscillatory shear (OS, ±5 dynes/cm^2^) using the cone-and-plate shear apparatus as previously described in our lab [32]. Early passages of iMAECs (passage # 6-10) and later passages of iMAECs (passage # 69-81) were also compared for their responses to shear stress. All shear stress studies were performed using complete growth medium for 24 h.

### Endothelial tube formation assay

Following 24 h shear exposure, as described above, iMAECs were trypsinized and resuspended in 2% fetal bovine serum (FBS)-containing DMEM and 20,000 cells/well were added to a 96-well flat bottom plate coated with growth factor-reduced Matrigel (BD Biosciences). Following culture for 20 h, tubule formation was observed using a phase contrast microscope at 10× magnification. Tubule length was quantified using NIH ImageJ software [33].

### Endothelial sprouting

Following 24 h shear exposure as described above, iMAECs were trypsinized, resuspended, and spotted at a density of 6 × 10^3^ cells were embedding in a 1:1 mixture of DMEM and Matrigel and gently placed on the bottom of a 6-well plate. After polymerization at room temperature for 20 min, 2 mL of complete media was added. Endothelial sprouting was determined 2 days later by bright-field microscopy and counting the cells outside the bead periphery.

### Scratch wound migration assay

A scratch-wound migration assay was performed using iMAECs previously exposed to either LS or OS for 24 h. Briefly, after shear, cell monolayers were scratched with a 200-μL pipette tip, media was replaced, and wounds were photographed at 0 and 6 hours [34]. NIH ImageJ software was used to quantify the closure of the wound over time by averaging six individual measurements of wound size for each wound at each time point. Results from three independent experiments performed in duplicate were pooled.

### Western blotting

Following exposure to shear, cells were washed three times with ice-cold phosphate-buffered saline (PBS) and lysed with radioimmunoprecipitation assay buffer (RIPA) as described previously [35]. The lysate was further homogenized by sonication. The protein content of each sample was determined by Bio-Rad DC assays. Aliquots of cell lysate (20 μg of protein) were then resolved by size on 10% SDS-polyacrylamide gels and subsequently transferred to a polyvinylidene difluoride membrane (Millipore). The membrane was incubated with a primary antibody overnight at 4°C, followed by incubation with an alkaline phosphatase-conjugated secondary antibody for 1 h at room temperature. Protein expression was detected using chemiluminescence, and the intensities of immunoreactive bands were determined via densitometry and the NIH ImageJ program [36]. Primary antibodies specific for KLF2, eNOS (BD Biosciences), β actin (Santa Cruz), VCAM-1 (Santa Cruz), Flk-1 (VEGF-R2, Santa Cruz), and Caveolin-1 (Santa Cruz) were used.

### Quantitative real time PCR (qPCR)

Total RNA of each sample was reverse transcribed into cDNA using SuperScript III and random primers (Invitrogen) as previously described [37]. Briefly, qPCR was performed on selected genes using Brilliant II SYBR Green QPCR Master Mix (Stratagene) with custom-designed primers using a real-time PCR system (ABI StepOne Plus). All qPCR results were normalized based on 18S RNA expression.

### Dihydroethidium (DHE) staining

iMAEC were stained in 2 μM DHE diluted in phosphate buffered saline for 30 minutes at 37°C. Cells were then fixed with 4% paraformaldehyde and mounted with DAKO mounting media and immediately imaged by fluorescence microscopy (Zeiss epi-fluorescent microscope).

## Results

### Morphology of cultured mouse aortic endothelial cells

**Figure 2:**
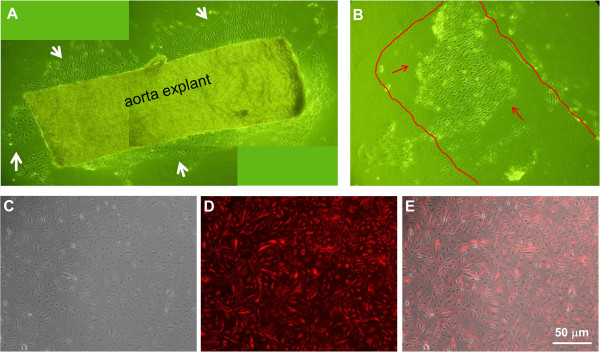
**Morphology of mouse aortic endothelial cells. (A)** Aortic explants were cultured on top of collagen gel beads for 4 days. EC grew and migrated out of the aorta piece. **(B)** EC grown on collagen gel beads without migration seems to keep their original elongated morphology. **(C-E)** iMAECs collected after cell sorting were cultured for 24 h and imaged (**C**: phase contrast, **D**: DiI-Ac-LDL staining by fluorescence microscopy, and **E**: merged image). Scale bar = 50 μm.

The summary of the MAEC isolation and immortalization procedure is shown in Figure 1. During explant culture on collagen gel beads, MAEC migrated out of the aortic explants and gradually covered the gel within 3-4 days (Figure 2A, white arrows). When aortic explants were removed after 3-5 days of culture on the collagen bead, patches of endothelial cells were observed (Figure 2B, red arrows), suggesting that these are endothelial cells that did not migrate out (the red line indicates where the aortic explant was before it was removed) (Figure 2B). Both the migrating cells and the endothelial patches were cultured in a 24-well plate and immortalized by the polyoma middle T antigen method, which specifically immortalizes ECs [29,30]. Immortalized cells were positively selected via G418 antibiotic over a 4 to 6 week period. To further ensure endothelial purity, we used a stringent gating strategy to collect only a portion of immortalized cells that were singlets and showed strong DiI-Ac-LDL fluorescence signal using HUVEC and RASM as positive and negative controls, respectively. Figure 2C-E show typical morphology of iMAEC obtained after the flow cytometric sorting using DiI-Ac-LDL.

### Characterization of iMAECs

**Figure 3:**
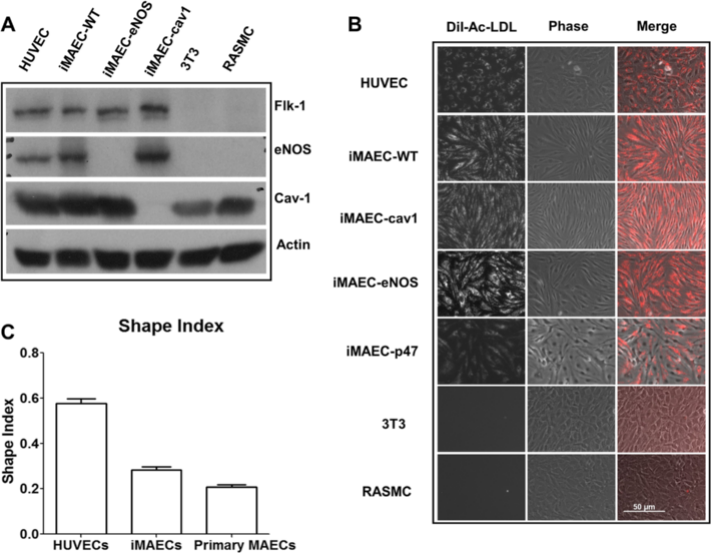
**Characterization of iMAEC lines by Dil-Ac-LDL staining.** iMAEC lines including wild-type (iMAEC-WT), Caveolin-1 knockout (iMAEC-cav1), eNOS knockout (iMAEC-eNOS), and p47^phox^ knockout (iMAEC-p47), were cultured. **(A)** Total cell lysates were collected from iMAEC lines or control cells including HUVEC, 3T3, and RASMC. Western blotting was performed using specific antibodies against Flk-1, eNOS and Cav-1. Actin serves as an internal loading control. **(B)** iMAECs were incubated with Dil-Ac-LDL (10 μg/mL) for 4 h, and images were taken by fluorescence microscopy. HUVEC served as positive control while 3T3 and RASMC served as negative controls. Scale bar: 50 μm. **(C)** Graph shows the cell shape index of HUVECs, iMAECs and primary MAECs. For cell shape index calculation, 25 cells were chosen randomly from each group and analyzed by ImageJ software. Data represent means ± standard error.

**Figure 4:**
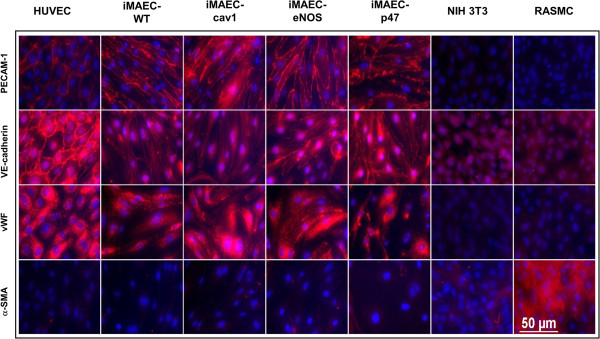
**Characterization of iMAEC lines by immunostaining.** iMAEC lines including iMAEC-WT, iMAEC-cav1, iMAEC-eNOS, and iMAEC-p47 were immunostained using the endothelial markers, PECAM-1, VE-Cadherin, and von Willebrand factor (vWF). Smooth muscle cell α-actin (α-SMA) was used as negative marker. HUVEC served as a positive control while 3T3 and RASMC were negative controls. Scale bar = 50 μm.

Using the described method, we developed several iMAEC lines, including wild-type (iMAEC-WT), Caveolin-1 knockout (iMAEC-cav1), eNOS knockout (iMAEC-eNOS), and p47^phox^ knockout (iMAEC-p47). The phenotype of these immortalized cells was then examined. First, we performed Western blotting to confirm the lack of protein expression in knockout cell lines. As expected, all iMAEC and HUVEC expressed Flk1, while 3T3 and RASMC did not. Next, caveolin-1 in iMAEC-cav1 or eNOS in iMAEC-eNOS did not express cavleolin-1 or eNOS protein, respectively (Figure 3A). To further confirm the identity of these cell lines, we studied Dil-Ac-LDL uptake, as endothelial cells internalize and degrade Ac-LDL 7-15 times more efficiently than smooth muscle cells [38]. As shown in Figure 3B, iMAEC showed homogeneous Dil-Ac-LDL uptake similar to primary HUVEC. As expected, 3T3 fibroblasts and rat aorta smooth muscle cells (RASMC) failed to uptake Dil-Ac-LDL, demonstrating the staining specificity of Dil-Ac-LDL to endothelial cells. We also determined the shape-index of iMAECs to compare the morphology of these cells to HUVECs and primary MAECs. We found that iMAECs are more spindle-shaped compared to the other ECs such as HUVECs (Figure 3C), which may reflect maintenance of the cytoskeletal organization under high shear stress environment of mouse aortic ECs *in vivo* as compared to the venous origin of HUVECs. Next, several endothelial-specific protein markers, PECAM-1, VE-Cadherin, and von Willebrand factor (vWF), were examined by immunocytochemistry. PECAM-1 and VE-Cadherin expression was localized as anticipated at the cell borders in iMAEC and HUVEC, as expected, but not in negative controls cells 3T3 and RASMC (Figure 4). Interestingly, iMAEC-cav1 cells showed a reduced junctional staining pattern for VE-cadherin. vWF was also observed in the cytosol of iMAECs and HUVECs, but not in the respective negative controls. In contrast, the smooth muscle cell specific maker, α-SMA, was positive only in RASMCs and negative in all ECs and the 3T3 cells, further confirming the specificity of iMAEC lines. These results demonstrate that iMAECs generated from wild-type and knockout mouse aortas retain key endothelial cell markers.

### iMAECs respond to shear stress

**Figure 5:**
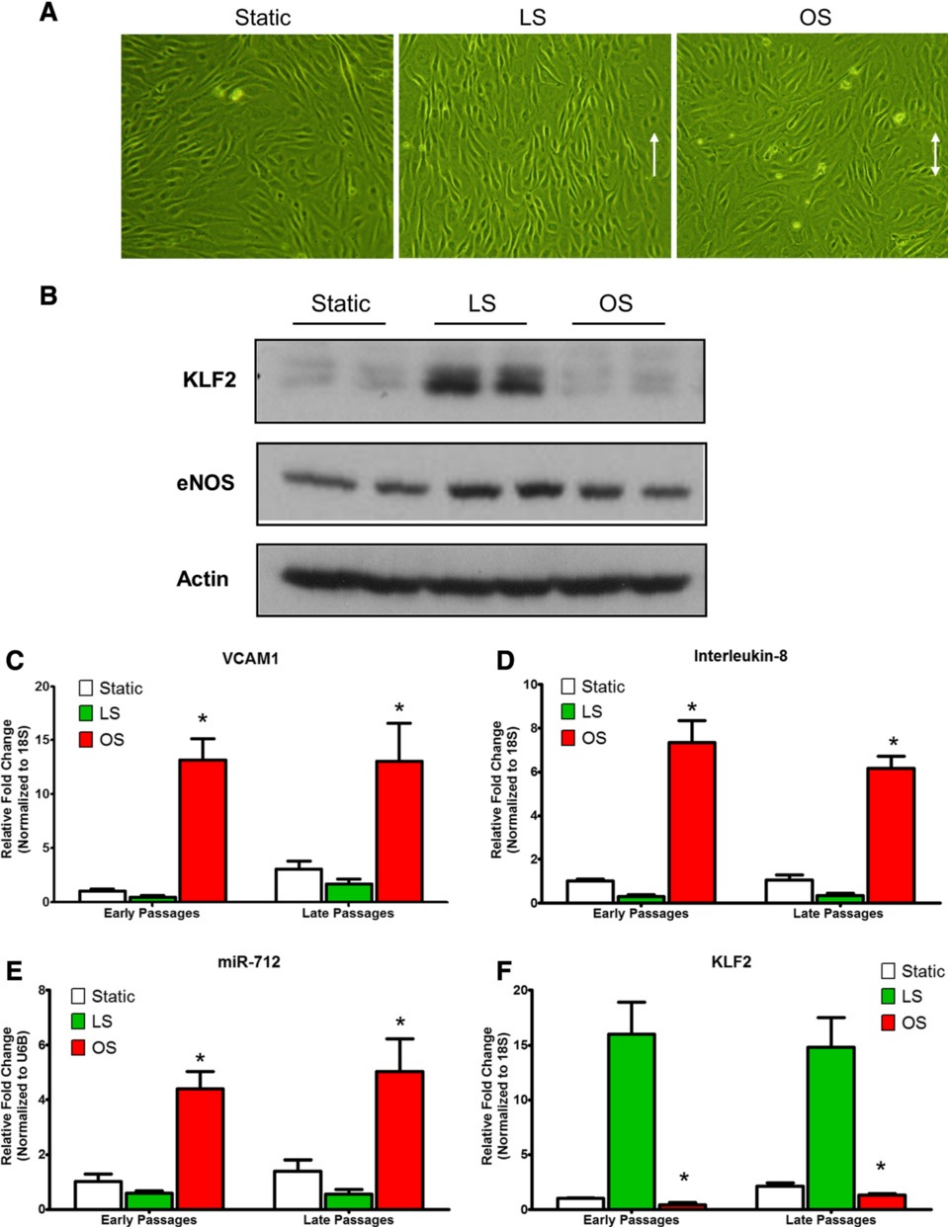
**iMAEC-WT maintains shear-sensitive endothelial phenotype.** iMAEC-WT were exposed to LS or OS or kept for static for 24 h. **(A)** iMAEC-WT aligned in the direction of the flow when exposed to LS but not OS or static control. Arrows indicate the imposed flow direction. **(B)** Total cell lysates were collected and protein level of KLF2 and eNOS was measured by Western blot. Data were shown as means ± standard error, n = 3. **(C-F)** Expression of shear-sensitive genes and microRNA under LS and OS for 24 h using early and late passages of iMAEC cells. iMAECs from early and late passages were subjected to either LS or OS for 24 h and expression of *VCAM1*, *KLF2* and *Interleukin-8***(C, D, F)** was determined by qPCR and normalized to 18S. **(E)** Graph shows the expression of shear-sensitive miRNA, miR-712 as determined by qPCR and normalized to RNU6B. Cells under static condition served as control. * p < 0.05 compared to respective LS controls.

To further test whether iMAECs behave similarly to primary human EC cultures, we tested morphological and gene expression changes of these cells in response to shear stress. Like other cultured ECs, iMAECs exposed to unidirectional laminar shear stress (LS) aligned in the direction of the imposed flow, while those cultured under the oscillatory shear (OS) or static conditions did not. Further, it is well-known that LS induces expression of *KLF2* and *eNOS* as compared to static culture or OS [39,40]. Consistent with previous reports [21], iMAEC-WT aligned to the direction of the flow in LS conditions but not in OS conditions after 24 h (Figure 5A). Further, LS increased KLF2 and eNOS protein expression (Figure 5B). These results demonstrate that iMAECs respond to shear stress in a manner consistent with other cultured ECs. We also followed the effect of shear stress on early passages of iMAECs (passage # 6-10) and compared to higher passage numbers of iMAECs (passage # 69-81) so as to confirm that the late passages of iMAECs respond to shear stress in the same fashion. We tested the expression of well-known shear sensitive genes, such as VCAM1, KLF2, KLF4, and Interleukin-8, as well as a shear-sensitive microRNA, miR-712 in response to either LS or OS for 24 h. We found that the expression of OS-induced VCAM1, Interleukin-8 and miR-712 showed a similar magnitude of fold-change in the early and late passages of iMAECs (Figure 5C-E). Also, the difference in expression of KLF2 between LS and OS was comparable to early passage cells (Figure 5E). These results indicate that iMAECs retain a functional endothelial phenotype and do not change significantly with multiple passages.

**Figure 6:**
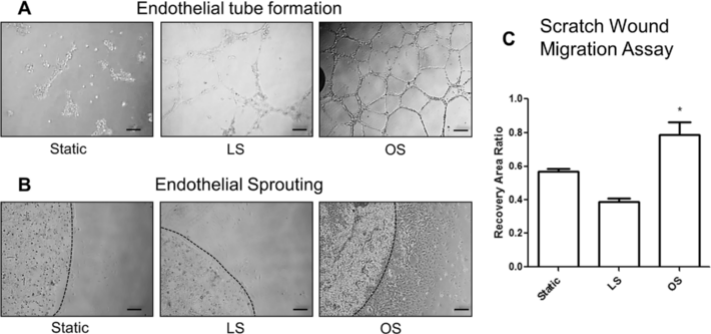
**Functional response of iMAECs to shear stress. (A)** Endothelial cell tube formation; **(B)** endothelial cell sprouting; and **(C)** endothelial cell scratch wound migration assay performed using iMAECs exposed to LS or OS for 24 h. Dotted black line in **B** denotes the periphery of the Matrigel bead for sprouting assay. Black scale bar = 200 μm. Cells under static condition were used a controls. Data shown as means ± standard error; n = 3; * p < 0.05.

Additionally, we have shown that OS induces endothelial tube formation and migration in various cultured ECs, including HUVEC [34,41,42]. Thus, we tested whether iMAECs respond to shear in a similar manner. As shown in Figure 6A-C, exposure of iMAECs to OS for 24 h induced endothelial tube formation, sprouting capability, and migration in the scratch wound-healing study, in comparison to LS and static conditions. These results suggest that iMAECs respond to shear stress in a manner similar to other cultured primary ECs.

**Figure 7:**
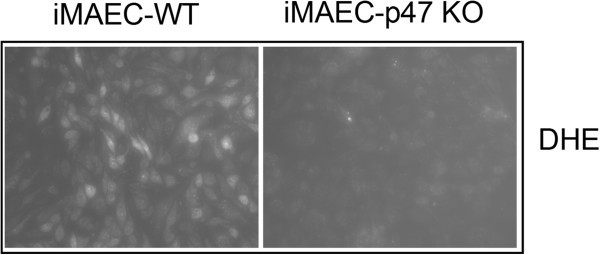
**VCAM-1 expression is elevated in iMAEC-eNOS while superoxide production is diminished in iMAEC-p47.** iMAEC-WT and iMAEC-p47 were stained with DHE (2 μM) for 30 min and images were acquired using fluorescence microscopy.

Next, we compared superoxide production between iMAEC-WT and iMAEC-p47phox Figure 7. Given that p47^phox^ is an important component of NADPH oxidases, which produce superoxide, lack of p47 ^phox^ in EC is expected to reduce the production of superoxide [21]. Indeed, superoxide production, as demonstrated by dihydroethidium (DHE) staining, was significantly lower in iMAEC-p47 cells as compared to iMAEC-WT, thus both confirming the knockout efficiency in these cells as well as the role of p47phox in superoxide production.

## Discussion

In this study, we developed an effective method to generate iMAEC lines that maintain an endothelial phenotype and respond to shear stress similar to other ECs, even after numerous passages. We also demonstrated functional differences between knockout and wild-type iMAEC lines in a gene-specific manner. For example, iMAEC-p47KO showed diminished production of superoxide. These results validate our MAEC isolation and immortalization protocol and provide a useful tool to study vascular biology. Given the vast array of transgenic mice, many unique iMAEC lines can be generated, expanded, and shared within research communities using this method.

Over the years, several groups have suggested different methods for the isolation and culture of primary MAEC [18,19,22-27]; however, the major issue of most protocols has been the difficulty in maintaining strict endothelial phenotype, as they are prone to transdifferentiation into non-endothelial phenotypes. In addition, it is difficult to obtain a large number of pure primary MAEC due to the small vessel size of mice. Moreover, characterization of primary endothelial cell cultures should be conducted regularly to confirm the lack of contamination or transdifferentiation. Previous reports have shown phenotypic change in cultured endothelial cells as a result of passaging [43]. It is difficult to maintain the phenotype of primary MAEC in sufficient amounts for a series of experiments without excessive time and effort. We compared iMAECs to HUVECs, which are the most widely used endothelial cells for *in vitro* experiments and are considered to be the “gold standard” for *in vitro* endothelial cell biology. Interestingly, iMAECs retained endothelial cell morphology similar to other endothelial cells derived from the high magnitude velocity environment of the arterial bed, which tend to remain more spindle-shaped and not cobblestone- shaped, which is the predominant morphology of cells derived from the reduced flow environment of the venous bed, such as HUVECs. Furthermore, in comparison to HUVECs, the expression of junctional proteins such as PECAM1 and VE-cadherin was relatively lower in the iMAECs, which is also in agreement to a previous report [44]. However, this did not impact EC permeability, as we recently demonstrated with iMAECs that EC permeability can be increased dramatically in response to either OS or miR-712 [41].

Importantly, immortalized cells are easily expandable and maintain an EC phenotype that for several months. It should also be noted that iMAEC lines only provide a model for studying vascular biology or disease *in vitro*, but may provide different responses when compared to primary cultures or *in vivo* studies. Given this caveat, researchers should be cautious when interpreting iMAEC-derived data and the results should be validated in primary cultured ECs and, more importantly, *in vivo*.

Explant culture of aortic tissue has been reported previously [18,23,25]. Most protocols used Matrigel as the base matrix for MAEC growth and migration [18,23,25]. Matrigel induces tube formation in ECs and has been widely used in angiogenesis studies. Matrigel may create an environment which stimulates cell proliferation and migration, potentially altering the phenotype of primary cells. Our method uses a collagen gel mixed with growth media, which has less of an effect on cellular phenotype, as demonstrated by our results To recapitulate, our results indicate that MAECs maintain their original morphology (Figure 2). Our modified method also provides a high yield of primary MAECs and they could be easily identified by their morphology, which is helpful when evaluating potential contamination of other cell types.

Immortalized mouse endothelial cells isolation from the embryo or brain in a variety of transgenic mice has been reported [31]. These studies demonstrated the need of transgenic iMAEC lines to address questions regarding the function of specific genes. To the best of our knowledge, this is the first report outlining a method to generate immortalized MAEC. As the origins of an EC, such as from an artery, vein, or microvessel, show different responses to stimuli, our method provides an adaptable protocol for developing EC lines from multiple locations.

## Conclusion

Here, we developed a simple method to generate iMAEC lines, which maintain EC phenotype and functional responses to physiologically relevant shear stresses similar to primary ECs. Therefore, iMAECs can be used for multiple passages to study endothelial biology *in vitro*, which reduces the batch-to-batch variation and the need for a large number of animals to isolate primary endothelial cells. This method can be applied to generate various knockout MAEC lines and used to study vascular biology and pathobiology.

## Competing interest

The authors declare that they have no competing interests.

## Authors’ contributions

CN, SK and CJA carried out the experiments, participated in the data analysis, and drafted the manuscript. HJ conceived the study, and participated in its design, edited the manuscript, secured funding, and supervised the overall research. All authors read and approved the final manuscript.
